# Lysine Acetyltransferase p300/CBP Plays an Important Role in Reproduction, Embryogenesis and Longevity of the Pea Aphid *Acyrthosiphon pisum*

**DOI:** 10.3390/insects11050265

**Published:** 2020-04-26

**Authors:** Phillipp Kirfel, Andreas Vilcinskas, Marisa Skaljac

**Affiliations:** 1 Fraunhofer Institute for Molecular Biology and Applied Ecology (IME), Branch for Bioresources, Ohlebergsweg 12, 35392 Giessen, Germany; phillipp.kirfel@ime.fraunhofer.de (P.K.); andreas.vilcinskas@agrar.uni-giessen.de (A.V.); 2 Institute for Insect Biotechnology, Justus-Liebig University of Giessen, Heinrich-Buff-Ring 26–32, 35392 Giessen, Germany

**Keywords:** KAT3, CREB-binding protein, RNA interference, senescence, life-history traits, *nejire*

## Abstract

CREB-binding protein (p300/CBP) is a universal transcriptional co-regulator with lysine acetyltransferase activity. *Drosophila melanogaster* p300/CBP is a well-known regulator of embryogenesis, and recent studies in beetles and cockroaches have revealed the importance of this protein during post-embryonic development and endocrine signaling. In pest insects, p300/CBP may therefore offer a useful target for control methods based on RNA interference (RNAi). We investigated the role of p300/CBP in the pea aphid (*Acyrthosiphon pisum*), a notorious pest insect used as a laboratory model for the analysis of complex life-history traits. The RNAi-based attenuation of *A. pisum* p300/CBP significantly reduced the aphid lifespan and number of offspring, as well as shortening the reproductive phase, suggesting the manipulation of this gene contributes to accelerated senescence. Furthermore, injection of p300/CBP dsRNA also reduced the number of viable offspring and increased the number of premature nymphs, which developed in abnormally structured ovaries. Our data confirm the evolutionarily conserved function of p300/CBP during insect embryogenesis and show that the protein has a critical effect on longevity, reproduction and development in *A. pisum*. The potent effect of p300/CBP silencing indicates that this regulatory protein is an ideal target for RNAi-based aphid control.

## 1. Introduction

Protein acetylation in eukaryotes is a major post-translational modification, in which acetyl coenzyme A acts as an acetyl group donor [1,2]. Although discovered as a unique modification of histones, acetylation marks are found on numerous non-histone proteins in all cellular compartments [3,4]. The acetylation of proteins regulates many processes, including gene expression, cell cycle progression, development and aging [3,4]. Acetylation affects the function of proteins by conferring a positive charge, which influences stability, enzymatic activity, subcellular localization and cross-talk with other protein modifications such as methylation [4].

The acetylation of proteins is regulated by the opposing activity of lysine acetyltransferases (KATs) and lysine deacetylases (KDACs) [3,5]. KATs catalyze the transfer of acetyl groups to a lysine residue, whereas KDACs remove these groups [5]. A fine balance between KAT and KDAC activities maintains normal biologic functions [6], so any disruption of this balance (caused naturally or triggered by the use of inhibitors) can severely affect physiology and development [7,8,9].

There is a remarkably diverse panel of highly conserved KDACs and KATs in many organisms [3]. Eleven groups of KDACs have been defined (KDAC1–KDAC11), whereas most KATs are assigned to three groups: the GCN5-related N-acetyltransferases (GNAT family); the p300/CREB-binding proteins (p300/CBP family); and the MOZ/Ybf2/Sas2/Tip60 (MYST) family [3]. The paralogs p300 (also known as EP300 and KAT3B) and CBP (also known as CREBBP, KAT3A and *nejire*) are often collectively described as p300/CBP [10,11].

In higher eukaryotes, p300/CBP is a key transcriptional co-regulator of basic cellular functions [10,11,12]. Evolutionary studies have identified p300/CBP as an essential enzyme that regulates the growth and development of multicellular organisms by controlling cell-to-cell signaling and morphogenesis [10,13,14,15]. Furthermore, p300/CBP is a major component of multiple signaling pathways [16,17,18,19]. More than 400 p300/CBP target proteins have been identified, leading to the acetylation of ~100 protein substrates [10]. The dysregulation of p300/CBP has been associated with several human diseases, including various forms of cancer [20]. In *Drosophila melanogaster*, the loss of p300/CBP activity causes severe embryonic defects [21,22]. In the cockroach *Blatella germanica* and the red flour beetle *Tribolium castaneum*, the knockdown of this gene revealed multiple roles in postembryonic development [23,24,25].

The pea aphid (*Acyrthosiphon pisum*) is a laboratory model for the analysis of plant–insect interactions and complex life-history traits and was the first hemipteran insect with a complete published genome sequence [26,27,28]. It is also a pest insect that damages crops by direct feeding and by vectoring numerous plant viruses [29]. A comprehensive set of *A. pisum* acetylation enzymes has been identified, some of which (KAT6B, KAT7, KAT14 and RPD3) regulate life-history traits such as longevity, development and reproduction [30,31]. Although histone acetylation may induce reproductive and wing morphology polyphenism in some aphids, no such correlation has been identified in *A. pisum* [30,32,33,34]. Despite the central role of p300/CBP as a transcriptional co-regulator, nothing is yet known about the function of this protein in aphids.

To address this knowledge gap, we investigated the role of p300/CBP in *A. pisum* by RNA interference (RNAi), a powerful approach for the functional analysis of genes in insects [35,36,37,38]. RNAi can also be used as a pest control strategy, by expressing double-stranded RNA (dsRNA) in crops or applying it as sprays [38,39,40,41,42,43,44,45]. We injected aphids with *p300/CBP* dsRNA and measured their fitness parameters to determine the effect of RNAi-mediated p300/CBP attenuation on longevity, reproduction and embryogenesis.

## 2. Materials and Methods

### 2.1. Aphid Rearing

*A. pisum* parthenogenetic clone LL01 was reared on 2–3-week-old bean plants (*Vicia faba* var. minor) in a KBWF 720 climate cabinet (Binder, Tuttlingen, Germany) with a 16-h photoperiod and a day/night temperature regime of 24/18 °C [37,46].

### 2.2. RNA Extraction, Target Gene Identification

**Figure 1:**
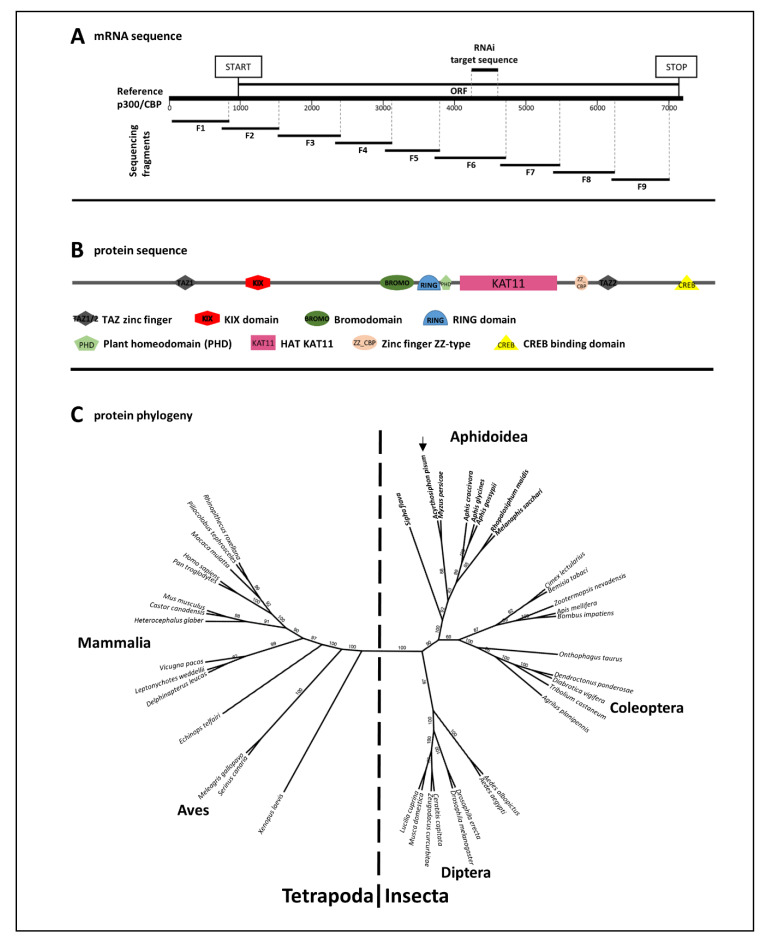
Characteristics of the *p300/CBP* sequences used in this study. (**A**) The *A. pisum p300/CBP* mRNA reference sequence (XM_008188962) is shown, the location of the open reading frame (ORF) as well as the RNAi target site is indicated. The orientation of the nine fragments obtained by cloning and Sanger-sequencing (Supplementary Fragments 1–9, Table S1) is depicted. These fragments were used for the assembly of *A. pisum* p300/CBP sequence. Our assembly contains the 5′-UTR and most of the open reading frame (ORF) including the start codon, but not the stop codon and 3′-UTR (**B**) Domain analysis of the p300/CBP protein sequence using Pfam and NCBI conserved domains databases. A complete set of p300/CBP typical domains was identified (**C**) Phylogeny of p300/CBP protein sequences. The tree was generated with RAxMl after MUSCLE alignment using amino acid sequence of *A. pisum* (black arrow/XP_003242232), *Sipha flava* (XP_025414151), *Myzus persicae* (XP_022176157), *Aphis craccivora* (KAF0769549), *Aphis glycines* (KAE9537982), *Aphis gossypii* (XP_027838800), *Rhopalosiphum maidis* (XP_026820749), *Melanaphis sacchari* (XP_025193438), *Cimex lectularius* (XP_014253865), *Bemisia tabaci* (XP_018901305), *Zootermopsis nevadensis* (XP_021919144), *Apis mellifera* (XP_026294862), *Bombus impatiens* (XP_012242677), *Onthophagus taurus* (XP_022908965), *Dendroctonus ponderosae* (XP_019756971), *Diabrotica vigifera* (XP_028149091) *Tribolium castaneum* (XP_008192360), *Agrilus planipennis* (XP_025830621), *Aedes albopictus* (XP_029711694), *Aedes aegypti* (XP_011493407), *Drosophila erecta* (XP_015011063), *Drosophila melanogaster* (NP_524642), *Ceratitis capitata* (XP_012155269), *Zeugodacus curcurbitae* (XP_028900992), *Musca domestica* (XP_011290197), *Lucilia cuprina* (XP_023298299), *Xenopus laevis* (NP_001088637), *Serinus canaria* (XP_009084782), *Meleagris gallopavo* (XP_010710456), *Echinops telfairi* (XP_004700331), *Delphinapterus leucas* (XP_022452845), *Leptonychotes weddellii* (XP_006729983), *Vicugna pacos* (XP_006207247), *Heterocephalus glaber* (EHB13435), *Castor canadensis* (JAV39871), *Mus musculus* (NP_808489), *Pan troglodytes* (NP_001231599), *Homo sapiens* (AAA18639), *Macaca mulatta* (NP_001253415), *Piliocolobus tephrosceles* (XP_023077657), *Rhinopithecus roxellana* (XP_010375568). Defined organism family clusters are indicated. GeneBank accession numbers and bootstrap values are shown within the tree.

We extracted total RNA from pools of 10 aphids using the NucleoSpin RNA kit (Macherey–Nagel, Germany) according to the manufacturer’s protocol. First-strand cDNA was synthesized from 100 ng RNA using the RevertAid first strand cDNA synthesis kit and dT primers (Thermo Fisher Scientific, Dreieich, Germany). We sequenced nine overlapping fragments covering the open reading frame (ORF) together with the 5′ untranslated region (5′-UTR) of the *A. pisum p300/CBP* mRNA (Figure 1, Table S1). The primers for sequencing were designed using Primer3 v4.1.0 (http://primer3.ut.ee/) and were based on the *A. pisum* sequence template from the NCBI database (XM_003242184). The overlapping p300/CBP fragments were cloned and sequenced as previously described [47].

### 2.3. Synthesis of dsRNA

We prepared dsRNA for RNAi experiments as previously described [30]. Briefly, the *A. pisum p300/CBP* mRNA sequence was used as a template and gene-specific RNAi primers including a 5′ T7 promoter were designed using Primer3 v4.1.0 and were purchased from Sigma-Aldrich (Germany). The dsRNA construct was designed to be 367 bp in length (GC content = 40%–60%) covering part of the ORF (Figure 1, Table S1). The construct was checked for off-targets by screening against the entire pea aphid genome, ensuring there were no overlaps >19 bp with other *A. pisum* genes. The PCR amplicon generated using the RNAi primers and cDNA template was cloned and sequenced as described above. The verified plasmid vector was used as a PCR template for the RNAi primers and the amplicon was excised from the gel and purified using the NucleoSpin Gel and PCR Clean-up kit (Macherey–Nagel). The purified PCR product was used to synthesize dsRNA with the Ambion MEGAscript T7 kit (Applied Biosystems, USA). The dsRNA was purified by isopropanol precipitation and washed with ethanol. The pellet was resuspended in 30–50 µL nuclease-free water and stored −20 °C. Primers and accession numbers for all *p300/CBP* sequences used in this study are listed in Table S1.

### 2.4. RNAi Injection Assays

In the RNAi experiments, 5-day-old aphids were injected using glass capillaries held on a M3301 micromanipulator (World Precision Instruments, USA). The aphids were injected laterally, between the mesothorax and metathorax, with 25 nL of the *p300/CBP* dsRNA (50, 250, 1000 or 3000 ng/µL) or GFP dsRNA as a control (3000 ng/µL). We injected a total of 200 aphids per treatment, comprising five biologic replicates of 40 aphids each. After injection, aphids were individually transferred to Petri dishes containing *V. faba* leaves on 1% agarose. Aphid survival and offspring production were monitored daily as previously described [30,47]. Developmental effects were determined by tracking the start of reproduction and the number of premature (dead) offspring (Figure S1), whereas the effect on reproduction was determined by tracking the total number of viable offspring and the number of viable offspring per day. Premature nymphs were not viable after eclosion and their antennae and legs remained folded [37,48]. Newly emerged nymphs were counted daily and removed. The Petri dishes and leaves were replaced every 5 days to ensure optimal conditions. To verify the observed effects on life history traits, we additionally injected two non-overlapping *p300/CBP* dsRNA fragments (3000 ng/µL) into 40 aphids each and monitored and analyzed the above-mentioned parameters for 14 days (Table S1, Figures S2 and S3).

We also measured the body weight (0-, 3- and 8-days post-injection), size (3 and 8 days post-injection) and color (3 and 8 days post-injection) of 40 individuals treated with 3000 ng/µL p300/CBP or GFP dsRNA. To record the size and color of the aphids, images were acquired using an MZ16FA stereomicroscope (Leica Microsystems, Wetzlar, Germany) and characterized using ImageJ v1.52.

To better understand the impact of p300/CBP silencing on *A. pisum* reproduction, we dissected ovaries from aphids injected with the highest concentration (3000 ng/µL) of *p300/CBP* or GFP dsRNA 10 days post-injection. The ovaries were stored in phosphate-buffered saline with 0.1% Tween-20 (PBST) and images of the dissected specimens were acquired as described above. We counted the total number of embryos in the ovaries but also the number of late-stage embryos (stage 18 or older, defined by the presence of visible eyes) and early stage embryos (stage 17 or younger, no visible eyes) [49].

The survival of aphids was also examined in the G1 generation to evaluate possible transgenerational silencing effects. The neonate G1 nymphs (40 per treatment or control) were collected 6 days after the injection of their mothers with 3000-ng/µL *p300/CBP* or GFP dsRNA. Aphid nymphs were individually transferred to Petri dishes with *V. faba* leaves and monitored for 2 weeks.

### 2.5. Quantitative PCR (qPCR)

Single aphids (n = 5) were collected 12 h post-injection (3000-ng/µL *p300/CBP* or GFP dsRNA) and RNA was extracted as described above. The RNA samples were treated with TurboDNase (Invitrogen, Germany) to ensure the complete removal of genomic DNA. We then purified the RNA using the NucleoSpin RNA extraction kit. The High-Capacity RNA to cDNA kit (Applied Biosystems) was used to generate cDNA according to the manufacturer’s recommendations. Gene-specific primers, designed using PrimerQuest (Integrated DNA Technologies, Coralville, IA, USA); http://eu.idtdna.com/PrimerQuest) and purchased from Sigma-Aldrich, were used in a 10-µl reaction to quantify the p300/CBP mRNA, comprising 10 µM specific primers, 5 µL 2x Power SYBR Green PCR Master Mix and 2 µL cDNA template (50 ng cDNA per reaction mixture). The StepOnePlus Real-Time PCR System (Applied Biosystems) was used with a primary denaturation step at 95 °C for 5 min followed by 40 cycles of 95 °C for 15 s and 60 °C for 60 s. We used three replicates for statistical analysis of target gene expression with REST2009 software [50]. The data were normalized against the ribosomal protein L32 (rpl32) gene in aphids. The sequences of all primers are provided in Table S1.

### 2.6. Bioinformatics and Data Analysis

Protein domains were predicted using the Pfam database [51] and the NCBI conserved domains database [52]. Alignments, sequence comparisons, and the assembly of p300/CBP gene fragments were achieved using Geneious v10.2.4 (https://www.geneious.com). Multiple sequence alignment was performed using MUSCLE [53], subsequently the phylogenetic tree was built using the RAxMl plug-in [54] for Geneious v10.2.4 with default parameters. The aphid fitness data were analyzed using IBM SPSS Statistics v26 (Armonk, USA). The threshold for statistical significance was set to *p* < 0.05 for all tests, except two-way analysis of variance (ANOVA) where the threshold was *p* < 0.001. The significance of survival, evaluated by Kaplan–Meier survival analysis, and the start of reproduction, visualized as bars, were calculated using the log-rank test. The total numbers of viable and premature offspring were analyzed using the Kruskal–Wallis test with Bonferroni correction for pairwise comparisons. The number of offspring per day was analyzed by two-way ANOVA, whereas body size, body color and body weight were evaluated using Student’s *t*-test.

## 3. Results

### 3.1. Genomic Sequence, mRNA Sequence and Protein Domain Analysis of A. pisum p300/CBP

The *p300/CBP* mRNA sequence from *A. pisum* clone LL01 was compared to the corresponding template sequence in the NCBI database (XM_003242184). The *A. pisum* p300-like template sequence is 7183 bp in length, whereas the size of our *p300/CBP* assembly was 6947 bp (Table S1). The assembly was produced by sequencing nine overlapping fragments of 800–1000 bp each (Table S1) and it comprises the 5′-UTR (928 bp) as well as the ORF including the ATG start codon (6019 bp). Based on the reference sequence from the NCBI (XM_008188962), the p300/CBP sequence obtained in this study is incomplete and does not include the last 134 nucleotides of the ORF, which features the stop codon and 3′-UTR. We also detected a few single nucleotide polymorphisms, but otherwise the assembly matched the *A. pisum* p300/CBP template (Figure 1A; Table S1).

Based on identified *p300/CBP* sequence in this study, a protein domain analysis revealed a distinctive set of p300/CBP domains that are conserved throughout known p300/CBP proteins of invertebrates and vertebrates. These include the KAT11 domain with acetylation activity, two TAZ-type zinc finger motifs necessary for DNA binding, a ZZ-type zinc finger with unknown function, a CBP-specific bromodomain responsible for interaction with acetylated lysine, a plant homeodomain, an atypical RING domain, as well as the characteristic KIX and CREB-binding protein-interaction domains (Figure 1B). The overall sequence is 59% identical at mRNA level and 49% identical at protein level to the *Apis melifera* p300/CBP sequences (55% at mRNA level and 42% at protein level to *Mus musculus* sequences). However, within the core catalytic region of the protein, spanning from the bromodomain to the downstream TAZ zinc finger domain, the sequence identity to the *A. melifera* protein sequence surpasses 80%, while the rest of the sequences appeared to be less conserved. In order to confirm that the sequence identified in this study indeed represents a p300/CBP homolog, we performed a phylogenetic analysis comparing p300/CBP protein sequences from several aphid species, other insects and vertebrates (Figure 1C). The identified *A. pisum* p300/CBP sequence clustered phylogenetically together in an aphid specific subgroup, closely related to other insect species, including other hemipterans (Figure 1C).

### 3.2. The Effect of RNAi-Mediated Attenuation of p300/CBP on Aphid Life-History Traits

**Figure 2:**
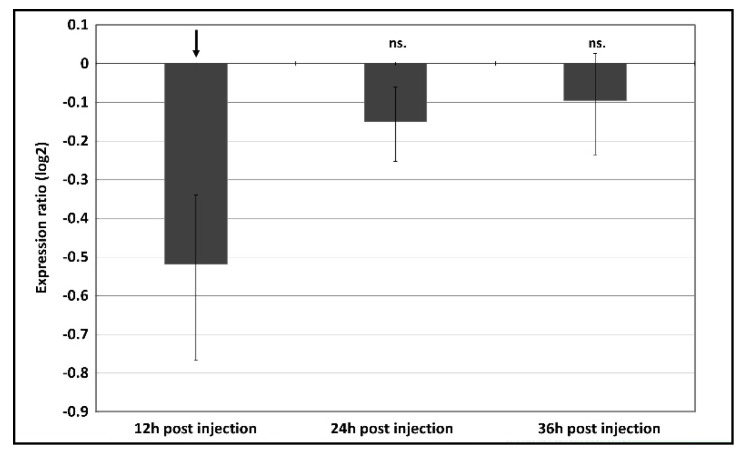
Expression ratio (log2) of p300 mRNA transcript determined using qPCR at 12 h, 24 h and 36 h post injection of gene-specific dsRNA in relation to the transcript expression in a GFP dsRNA treated control group. A negative expression ratio indicates downregulation, the expression was normalized against reference gene *rpl32*. Arrow indicates a significant variation of gene expression as calculated by REST analysis (*p* = 0.015). ns—not significant.

Following the injection of dsRNA, a significant decrease of *p300/CBP* transcripts was confirmed 12 h post injection using qPCR (~30% reduction; *p* = 0.015) (Figure 2).

**Figure 3:**
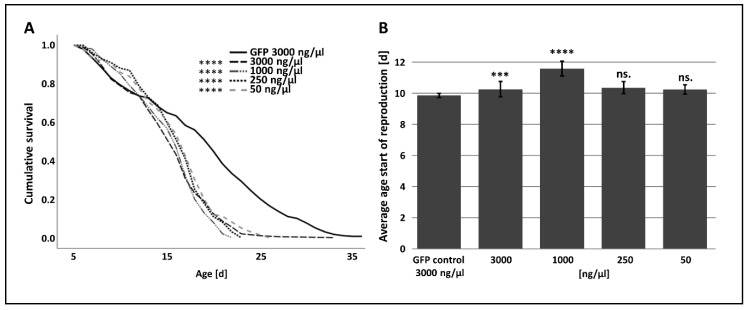
Life-history parameters following the injection of p300/CBP dsRNA in *A. pisum*. (**A**) Survival and (**B**) start of reproduction were monitored after the injection of 3000-, 1000-, 250- or 50-ng/µL dsRNA. Per treatment a total of 200 individuals were injected. Data were analyzed using a log-rank test. Statistical significance is indicated as follows: *** *p* < 0.001, **** *p* < 0.0001, ns—not significant.

The aphid survival did not differ significantly between the *p300/CBP* dsRNA and GFP dsRNA control group during the first ~5 days post-injection. However, the overall lifespan of aphids injected with *p300/CBP* dsRNA was severely reduced (Table 1 and Table S2, Figure 3A).

The p300/CBP dsRNA treatment showed the strongest impact 15 days post-injection when only ~10% of aphids survived. After 20 days, there were very few survivors. In the GFP dsRNA control group, ~50% of the aphids remained alive 15 days post-injection, and 20% remained alive after 20 days (Table 1, Figure 3A).

**Figure 4:**
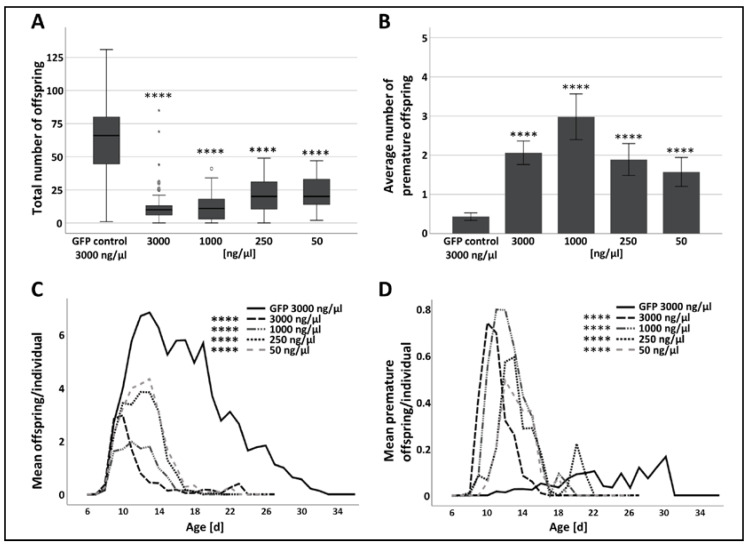
Reproduction parameters following the RNAi-mediated attenuation of p300/CBP in *A. pisum*. (**A**) The total number of offspring, (**B**) average number of premature offspring, (**C**) number of viable offspring per day and (**D**) number of premature offspring per day were monitored after the injection of 3000-, 1000-, 250- or 50-ng/µL *p300/CBP* dsRNA. 200 individuals per treatment were injected. To identify significant differences, we used (**A**,**B**) the Kruskal–Wallis test followed by Bonferroni corrections for pairwise analysis (**** *p* < 0.0001) or (**C**,**D**) two-way ANOVA (**** *p* < 0.000001).

The injection of large amounts of p300/CBP dsRNA (3000 ng/µL and 1000 ng/µL) induced a mild but significant delay to the start of the reproduction (Table S3, Figure 3B). The total number of offspring was significantly reduced in all four p300/CBP treatment groups compared to the GFP control, with 82% reduction in the 3000 ng/µL group, 82% reduction in the 1000 ng/µL group, 65% reduction in the 250 ng/µL group and 63% reduction in the 50 ng/µL group (Table S4, Figure 4A).

Further analysis revealed that the number of offspring per individual per day was also substantially lower in the *p300/CBP* dsRNA groups and was reduced in a concentration-dependent manner (Table S5, Figure 4C). Remarkably, the reproductive phase of aphids injected with *p300/CBP* dsRNA was much shorter (7–10 days) compared to the control group (up to ~25 days) (Figure 4C). The injection of p300/CBP dsRNA induced a significant increase in the number of premature nymphs throughout the reproductive phase (Tables S6 and S7, Figure 4B,D). The appearance of premature offspring indicated that p300/CBP plays a key role in aphid embryogenesis and/or eclosion.

To further verify that the observed effects are due to the suppression of p300/CBP and to minimize the possibility of off-target effects, we injected two additional, non-overlapping *p300/CBP* dsRNA constructs in the highest concentration (3000 ng/µL). The impact on life history traits of the injection of all three dsRNA fragments were comparable (Figure 3, Figure 4 and Figure S3).

**Figure 5:**
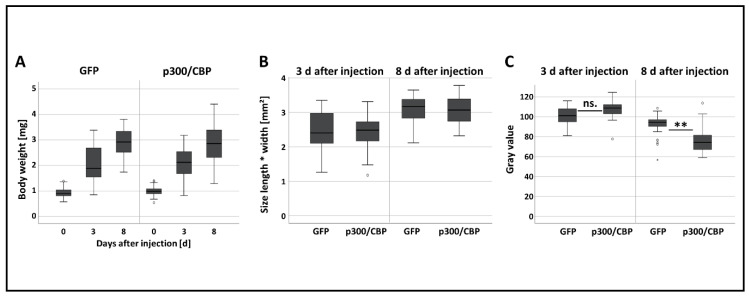
Life-history parameters following the RNAi-mediated attenuation of p300/CBP in *A. pisum*. (**A**) Bodyweight, (**B**) body size and (**C**) body color were determined on days 3 and 8 after treatment with 3000 ng/µL *p300/CBP* or *GFP* dsRNA. Aphid weight and size did not differ significantly between the p300/CBP treatment and GFP control groups. Data were analyzed using Student’s *t*-test. Statistical significance is indicated as follows: ** *p* < 0.01, ns—not significant.

The RNAi-mediated manipulation of p300/CBP did not induce changes in body weight, size or polyphenism (Tables S8 and S9, Figure 5A,B). However, the aphids injected with *p300/CBP* dsRNA became significantly darker in color 8 days post-injection, even though there were no significant differences 3 days post-injection (Table S10, Figure 5C).

### 3.3. Effect of RNAi-Mediated Knockdown of p300/CBP on Aphid Embryogenesis and the Transgenerational Silencing Effect in the G1 Generation

**Figure 6:**
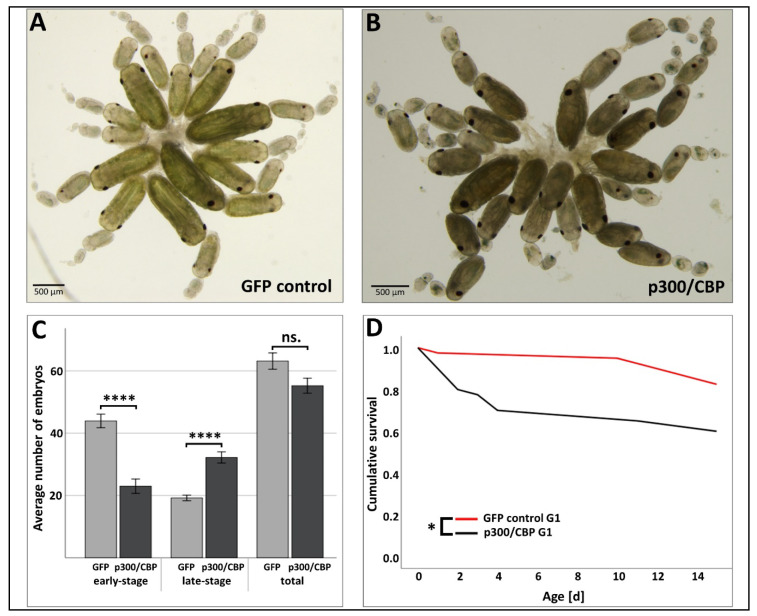
Effect of RNAi-mediated p300/CBP mitigation on the development and survival of the G1 generation of *A. pisum* mothers injected with dsRNA. Ovaries were dissected from aphids 10 days post-injection with (**A**) *GFP* control dsRNA or (**B**) *p300/CBP* dsRNA. (**C**) The distribution of early stage embryos (up to stage 17, no visible eyes) and late-stage embryos (stage 18 and beyond, visible eyes) differed significantly between the treatments. Ovaries from aphids treated with *p300/CBP* dsRNA contained significantly fewer early stage embryos (*p* < 0.0001) and significantly more late-stage embryos (*p* < 0.0001) than the *GFP* control group, but there was no difference in the total number of embryos. (**D**) The survival of G1 aphids from the *p300/CBP* dsRNA treatment group was compared to the GFP control group for 2 weeks. The survival of aphids in the *p300/CBP* dsRNA treatment group was significantly reduced compared to the control group (*p* < 0.05). The number of embryos was analyzed using Student’s *t*-test. Survival data were evaluated using Kaplan–Meier statistics and comparisons between the treatment and control were based on log-rank tests. Statistical significance is indicated as follows: * *p* < 0.05, **** *p* < 0.0001, ns—not significant.

In order to better understand the impact of p300/CBP dsRNA injection on *A. pisum* embryogenesis, we dissected ovaries from individuals in the p300/CBP treatment and GFP control groups. Ovaries dissected from aphids treated with *p300/CBP* dsRNA contained a greater number of late-stage embryos 10 days post-injection, and the tissue structure of ovaries was very fragile and highly susceptible to ruptures (Figure 6A–C). In contrast, ovaries dissected from aphids injected with the *GFP* dsRNA control had a normal tissue structure and contained embryos spanning all developmental stages (Figure 6A–C).

We monitored the survival of the viable offspring (G1 generation) of the aphids treated with *p300/CBP* dsRNA to investigate the potential for transgenerational effects. We found that the survival rate among the offspring of mothers injected with *p300/CBP* dsRNA was significantly lower than peers from the control group, whose mothers were injected with *GFP* dsRNA (Figure 6D). Although the survival of the G1 generation from the *p300/CBP* dsRNA group was affected, there were no differences in offspring count or viability between the treatment and control groups (data not shown).

## 4. Discussion

p300/CBP is one of the most entangled transcriptional co-regulators with hundreds of interaction partners found in a variety of multicellular organisms, from invertebrates to vertebrates [10,24,55]. In the pea aphid, p300/CBP has been identified previously in silico [31]. We further extended the characterization of aphid p300/CBP mRNA by cloning and sequencing (Figure 1 and Figure S2, Table S1). Subsequently, we were able to confirm the extensive subset of typical p300/CBP protein domains known from other species [10,55]. The core catalytic acetylation domain seems to be highly conserved and this is demonstrated by the similarities of p300/CBP in bees and aphids [10]. The phylogenetic analysis of p300/CBP protein sequences of a wide range of species, showed a clear separation of vertebrate and insect p300/CBP sequences, however, it endorsed a close relationship of all analyzed p300/CBP sequences including the one identified in our study (Figure 1C).

The analysis of insect p300/CBP protein function has focused mostly on *D. melanogaster*, but more recently the role of this protein has been investigated in *T. castaneum*, *B. germanica*, *Camponotus floridanus* and *Bombyx mori* [21,23,24,25,56,57,58,59]. We have expanded the scope of these experiments to include the first hemipteran model, *A. pisum*. In common with the studies involving *T. castaneum* and *B. germanica*, we used RNAi experiments to investigate the functions of p300/CBP while also evaluating its potential as a target for RNAi-mediated pest control in aphids.

The attenuation of p300/CBP in *A. pisum* resulted in severe fitness costs, supporting its role as a regulator of fundamental cellular processes as previously reported for other insects [23,24]. The injection of *p300/CBP* dsRNA significantly reduced the lifespan of the aphids as well as substantially shortening the reproductive phase, leading to the production of fewer offspring compared to peers in the *GFP* dsRNA control group (Table 1; Figure 3A and Figure 4A,C). The inhibition of p300/CBP has previously been shown to promote senescence in human cells, prevent lifespan extension in *Caenorhabditis elegans* and increase the likelihood of apoptosis in the insect cell line BmN [60,61,62]. These findings are also in agreement with the low levels of p300/CBP found in aging mice [63]. A decline in longevity and fecundity is naturally correlated with biologic aging in aphids [64]. Therefore, the fitness costs observed in *A. pisum* could be an indication of senescence induced by the manipulation of p300/CBP.

The depletion of p300/CBP unexpectedly inhibited food intake in *B. germanica*, leading to the production of underdeveloped nymphs, and reduced foraging behavior in the ant *C. floridanus* [23,58]. Although the mitigation of p300/CBP in aphids delayed the start of reproduction, their body weight and body size were unaffected (Figure 3B and Figure 5A,B). Furthermore, we observed no obvious changes in feeding behavior that would indicate a correlation between developmental costs and impaired nutrition. In *B. germanica*, p300/CBP silencing modulated the expression of genes encoding enzymes involved in gluconeogenesis and lipidogenesis [23]. Furthermore, p300/CBP dependent hyperacetylation stabilizes several nutritional storage proteins in *B. mori* [59]. It would therefore be interesting to investigate in detail whether the silencing of p300/CBP in aphids affects nutritional metabolism, but also feeding behavior.

The attenuation of p300/CBP in aphids caused an increase in the occurrence of premature (dead) offspring (Figure 4B,D), agreeing with the important role of p300/CBP during embryogenesis in *D. melanogaster*, *C. elegans* and mice [21,65,66]. This demonstrates that p300/CBP has an evolutionarily conserved function in eukaryotes. In addition to lethality, the loss of p300/CBP causes severe defects in *D. melanogaster* embryos including the absence of the head, thorax and cuticular structures [21,22]. We observed no such obvious defects in the aphid embryos produced by mothers in the *p300/CBP* dsRNA treatment groups, but the tissue structure of ovaries was very fragile and highly susceptible to ruptures (Figure 6A,B). The lack of tissue integrity is likely to reflect the role of p300/CBP in cell-to-cell communication during organ development and morphogenesis [13]. Interestingly, obstructing p300/CBP in aphids not only increased the number of premature offspring, but also triggered the retention of embryos by their mothers (Figure 4C,D). The dissection of aphids injected with p300/CBP dsRNA revealed ovaries that contained a large number of late-stage embryos, whereas ovaries from aphids in the GFP control group contained embryos spanning all developmental stages (Figure 6A–C). Embryo retention is not a well-understood phenomenon in aphids, but it can be associated with factors ranging from disrupted embryogenesis to biologic aging [64].

The p300/CBP protein also has an important role in post-embryonic development and metamorphosis in species such as *B. germanica* and *T. castaneum* [23,24]. The knockdown of p300/CBP in *T. castaneum* suppressed the expression of more than 1300 genes encoding transcription factors and other regulatory proteins. This had numerous physiological effects, including the enhancement of melanization in the midgut as a consequence of changes in innate immunity, pigmentation and metabolism [24]. Hyperpigmentation (dark green) was observed in aphids injected with *p300/CBP* dsRNA, but this affected the whole body rather than restricted tissues (Figure 5C). Follow-up studies should investigate in detail any correlations between the disregulation of p300/CBP and components of aphid immunity such as the phenoloxidase system.

In the grain aphid (*Sitobion aveane*), RNAi-mediated silencing of the *shp* gene was shown to reduce the quantity of saliva sheath protein produced for up to seven generations [67]. Therefore, we investigated the potential transgenerational effects of p300/CBP silencing in *A. pisum*. We observed a higher mortality rate during the first few days after birth in the G1 generation of *A. pisum* from the *p300/CBP* dsRNA group, whereas aphids from the control group were unaffected (Figure 6D). We did not observe any overt morphologic aberrations in viable G1 aphids, but this does not rule out a key role for p300/CBP during post-embryonic development, as previously reported for *B. germanica* and *T. castaneum*. Future studies should investigate whether p300/CBP is involved in the post-embryonic development of aphids, perhaps through regulation of juvenile hormones and ecdysteroids, as previously shown for cockroaches and beetles [23,24,25].

Finally, we analyzed the expression of the endogenous *A. pisum p300/CBP* gene following the injection of *p300/CBP* dsRNA. We anticipated that *p300/CBP* dsRNA would have a direct impact on the expression of the target gene at the posttranscriptional level, but RNAi also has the potential to modify transcription by means of feedback regulation to maintain chromatin homeostasis [68]. Although the attenuation of p300/CBP caused remarkable effects on *A. pisum* life-history traits, we observed only a small change in endogenous *p300/CBP* mRNA levels (~30% reduction) 12 h post-injection (Figure 2). Given the variable efficiency of RNAi in hemipteran species, RNAi effects can be observed even if there is a low detectable impact on target gene expression [30,35,69,70]. This may be due to transient silencing that escapes detection—or may reflect the costs associated with dsRNA degradation in the hemolymph of hemipteran insects [35,71]. In addition, it has been shown in *D. melanogaster* that microRNA mediated gene silencing can occur via multiple pathways and can act through translational instead of transcriptional repression [72,73,74]. Hence, to verify the hypothesis of a specific dsRNA mediated dysregulation of p300/CBP and the subsequent deterioration of aphid fitness, we injected two additional non-overlapping dsRNA constructs targeting p300/CBP. The treatments resulted in the same, strong developmental as well as lifespan aberrations (Figure 3, Figure 4 and Figure S3), minimizing the chances that the observed phenotypic alterations are off-target effects. Besides the moderate reduction of p300/CBP transcripts it is possible that protein levels were lower in aphids due to the inhibition of translation, which would require the quantitation of p300/CBP by western blot or similar methods [75,76]. Follow up studies need to clarify the importance of translational repression of dsRNA mediated gene silencing in aphids, which could also add another layer of complexity to the anyway challenging RNAi mediated gene repression in aphids.

## 5. Conclusions

In conclusion, we have shown that the RNAi-mediated inhibition of p300/CBP has a remarkably potent negative impact on life-history traits in *A. pisum*, significantly contributing to our understanding of p300/CBP as a universal transcriptional co-regulator in insects [24]. It would be valuable to investigate the function of p300/CBP in more detail by RNAi-mediated silencing followed by differential gene expression analysis to identify p300/CBP target genes in *A. pisum* as previously shown in beetles [24]. The RNAi-mediated control of aphids and other pest insects has already been demonstrated by the development of transgenic crops expressing dsRNA [38,39,45,67,77]. The transgenic approach may not be efficient for every pest and every crop, therefore growing evidence supports the utilization of dsRNA spray formulations as next-generation insecticides. The targeting of p300/CBP in this manner could provide an efficient and environmentally sustainable approach to reduce the agricultural damage caused by aphid pests.

## Figures and Tables

**Table 1:** Survival frequency [%] of aphids 5-, 10-, 15- and 20-days post-injection.

Treatment	Post-Injection Survival Frequency [%]			
	After 5 Days	After 10 Days	After 15 Days	After 20 Days
GFP 3000 ng/µL	79	65	45	20
p300/CBP 3000 ng/µL	79	51	13	1
p300/CBP 1000 ng/µL	85	57	8	0
p300/CBP 250 ng/µL	88	60	11	0
p300/CBP 50 ng/µL	85	61	12	3

## Supplementary Materials

The following are available online at https://www.mdpi.com/2075-4450/11/5/265/s1, Figure S1: Nymphs of p399/CBP dsRNA treated mothers, Figure S2: The *A. pisum p300/CBP* mRNA sequence from clone LL01 with RNAi target sides, Figure S3: Life history parameters following the RNAi-mediated silencing of p300/CBP, Table S1: Primer sequences used in this study, Table S2: Statistical analysis of RNAi data for survival compared to the GFP control, Table S3: Statistical analysis of RNAi data for start of reproduction compared to the GFP control, Table S4: Statistical analysis of RNAi data for average number of offspring compared to the GFP control, Table S5: Reproductive parameters evaluated during the RNAi experiments including viviparous offspring determined by two-way ANOVA, Table S6: Statistical analysis of RNAi data for average number of premature offspring compared to the GFP control, Table S7: Reproductive parameters evaluated during the RNAi experiments including premature offspring determined by two-way ANOVA, Table S8: Statistical analysis of RNAi data for body weight [mg] compared to the GFP control (dsRNA concentration = 3000 ng/µL), Table S9: Statistical analysis of RNAi data for body size (length × width)(mm^2^) compared to the GFP control (dsRNA concentration = 3000 ng/µL), Table S10: Statistical analysis of RNAi data for body color (grayscale) compared to the GFP control (dsRNA concentration = 3000 ng/µL), Sequence of p300, Supplementary Fragments: *A. pisum* p300/CBP mRNA, with primer positions underlined and indicated in bold (see Table S1).
