# Supplementary material for: Lysine Acetyltransferase p300/CBP Plays an Important Role in Reproduction, Embryogenesis and Longevity of the Pea Aphid Acyrthosiphon pisum
Source: Insects. 2020 Apr 26;11(5):265. doi: 10.3390/insects11050265 (PMC7290403; doi:10.3390/insects11050265)
Supplement: Supplementary file 1 [file insects-11-00265-s001.pdf]

Supplementary material

Article

Lysine Acetyltransferase p300/CBP Plays an Important Role in Reproduction, Embryogenesis and Longevity of the Pea Aphid *Acyrthosiphon pisum*

Phillipp Kirfel [1], Andreas Vilcinskas [1,2] and Marisa Skaljac [1,*]

[1] Fraunhofer Institute for Molecular Biology and Applied Ecology (IME), Branch for Bioresources, Ohlebergsweg 12, 35392 Giessen, Germany; phillipp.kirfel@ime.fraunhofer.de

[2] Institute for Insect Biotechnology, Justus-Liebig University of Giessen, Heinrich-Buff-Ring 26-32, 35392 Giessen, Germany; andreas.vilcinskas@agrar.uni-giessen.de

* Correspondence: marisa.skaljac82@gmail.com; Tel.: +49-641-9937747

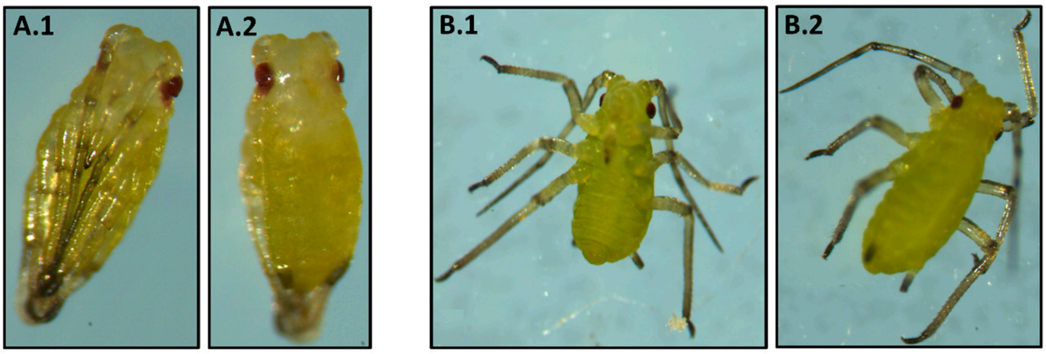

Figure S1. Nymphs of *p300/CBP* dsRNA treated mothers. A prematurely born nymph (A) in ventral (A.1) and dorsal (A.2) view. The legs and antenna remain unfolded and the nymphs die after eclosion. In comparison, a viable nymph (B) in ventral (B.1) and dorsal (B.2) view.

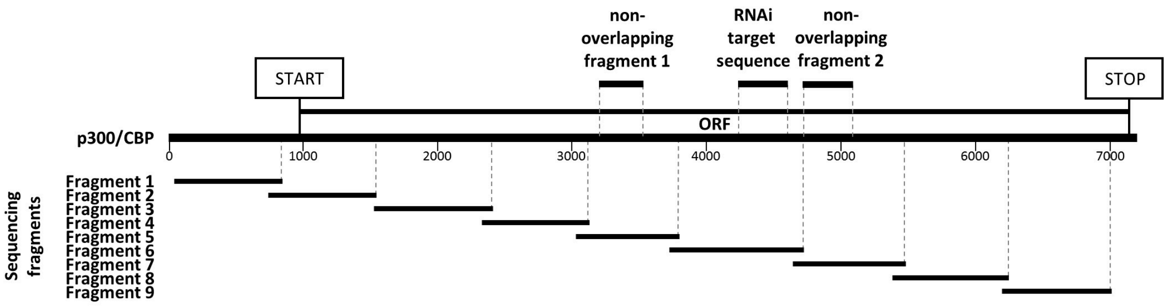

Figure S2. The *A. pisum p300/CBP* mRNA sequence from clone LL01 contains the 5′-UTR, start codon and most of the open reading frame (ORF), but the stop codon and 3′-UTR were not represented in our assembly (Tab. S1). The RNAi target site is also shown. Additional RNAi target sides used in this study are indicated as non-overlapping fragments 1 and 2.

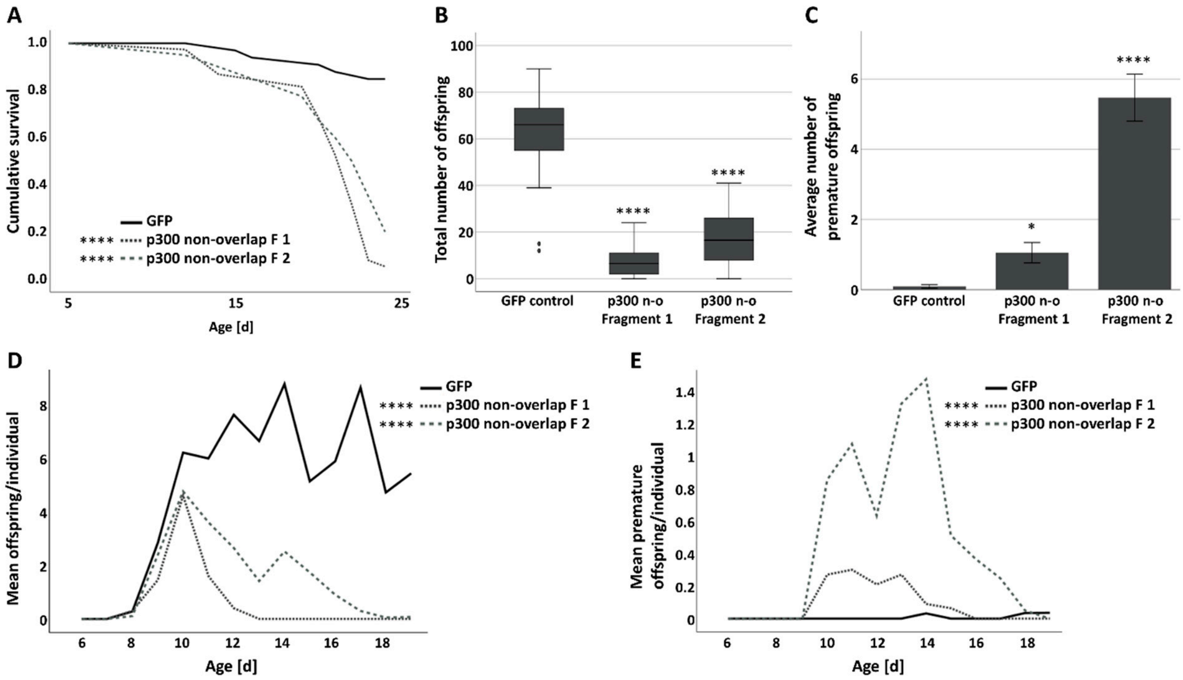

Figure S3: Life history parameters following the RNAi-mediated silencing of p300/CBP with the additionally tested non-overlapping fragments (called p300 non-overlap F1 and p300 non-overlap F2) as specified in Figure S2. We injected 40 individuals each with 25nl of a 3000 ng/µl solution. (A) survival, (B) total number of offspring, (C) average number of premature offspring, (D) number of viable offspring per day and (E) number of premature offspring per day is shown. Significances were tested using (A) the log-rank test, (B,C) the Kruskal-Wallis-test followed by Bonferroni corrections for pairwise analysis (*p < 0.05, ****p < 0.0001) and (D,E) two-way-ANOVA (*****p*< 0.000001).

Table S1. Primer sequences used in this study.

	Primer name	Accession No. and residues (nt) sequences	Sequence	Frag. size (bp)	purpose
**Fragment1**	seq_AP_p300_fwd_1	MN734788	5'CTG ACC TTG GAC CGT GAG TT3'	807	sequencing
	seq_AP_p300_rev_1	nt1 – nt807	5'CAA ATT GAG AAA CAA AAT AGT TAA ACA3'		
**Fragment2**	seq_AP_p300_fwd_2	MN734788	5'AAT CAG CCT CCG ACT TAG CA3'	856	sequencing
	seq_AP_p300_rev_2	nt700 – nt1555	5'CTG GTT GCG AAT TTG TTG TG3'		
**Fragment3**	seq_AP_p300_fwd_3	MN734788	5'AGC AAC AAC AAA ACG GAC CT3'	887	sequencing
	seq_AP_p300_rev_3	nt1485 – nt2371	5'TAC CTG GGG TTG TGA ATG TG3'		
**Fragment4**	seq_AP_p300_fwd_4	MN734788	5'CTC AAA TGC CAC ATC CTC CT3'	801	sequencing
	seq_AP_p300_rev_4	nt2286 – nt3086	5'TTG GAA AAT GTA CCC TGT CTG A3'		
**Fragment5**	seq_AP_p300_fwd_5	MN734788	5'TCA ATT CAA CCC AAT CAG CA3'	769	sequencing
	seq_AP_p300_rev_5	nt2990 – nt3758	5'CCA TTT TCT TTG AAC TTG AAC CA3'		
**Fragment6**	seq_AP_p300_fwd_6	MN734788	5'AAC ACC GTC GGC ATC TGT GA3'	1002	sequencing
	seq_AP_p300_rev_6	nt3685 – nt4687	5'AGC AAC TTC ACC AGC GCC TG3'		
**Fragment7**	seq_AP_p300_fwd_7	MN734788	5'ATT GGC TAT AAC AAA ATT AGG TAC G3'	839	sequencing
	seq_AP_p300_rev_7	nt4600 – nt5438	5'TGC TCT TTC TCT GGC TGG AC3'		
**Fragment8**	seq_AP_p300_fwd_8	MN734788	5'GAA GTT GAA ATT GGC CCT GA3'	865	sequencing
	seq_AP_p300_rev_8	nt5351 – nt6215	5'GCT GTG CTT GTT GCA GTC TT3'		
**Fragment9**	seq_AP_p300_fwd_9	MN734788	5'TGT CCA GTG TTG TTC TGT CCA3'	811	sequencing
	seq_AP_p300_rev_9	nt6137 – nt6947	5'GGA ACA TTT GCC TTT GTC GT3'		
**Assembly**	Fragment1-Fragment9	MN734788			Assembling
**Reference sequences**	Protein	XP_008187184			
	Nucleotide	XM_008188962			
	RNAi_AP_p300_fwd		5' **T7** G TCT TGG TTA TTG CTG TGG TCG T3'	367	RNAi
	RNAi_AP_p300_rev		5' **T7** T CAC CGC GCT TCT TCA AAC AGT3'		
	RNAi_AP_p300_fwd_1		5' **T7** AGC CAC CTG TCC CAA GTC CA3'		RNAi non-overlapping Fragment 1
	RNAi_AP_p300_rev_1		5' **T7** GGT ACA CTT GTA GTT GAT GGC AC3'		
	RNAi_AP_p300_fwd_2		5' **T7** GCG CTG GTG AAG TTG CTA TAC G3'		RNAi non-overlapping Fragment 2
	RNAi_AP_p300_rev_2		5' **T7** GCA TGC CCA AAT GTG AGC CA3'		
	Rpl32_F		5'AGT ATC GCC CAA CAA TTA TCA3'		qPCR reference gene [1]
	Rpl32_R		5'CTT GAA TCG TCT TCG GAC T3'		
	qPCR_AP_p300_fwd4		5'GTT GCG AAC TCT AGG TCA GAA TA3'		qPCR target gene
	qPCR_AP_p300_rev4		5'GCT GTT GCA TTT GTT GTT GTT C3'		

Table S2. Statistical analysis of RNAi data for survival compared to the GFP control.

Treatment	Mean survival [d]	Standard error [d]	Chi-square	Significance
**Control (GFP)**	13.88	0.44		
**p300/CBP 3000 ng/µl**	10.23	0.34	63.95	p<0.0000
**p300/CBP 1000 ng/µl**	10.26	0.39	49.03	p<0.0000
**p300/CBP 250 ng/µl**	10.93	0.40	38.21	p<0.0000
**p300/CBP 50 ng/µl**	11.08	0.47	30.65	p<0.0000

Table S3. Statistical analysis of RNAi data for start of reproduction compared to the GFP control.

treatment	Mean start [d]	Standard error [d]	Chi-square	significances
**Control (GFP)**	4.93	0.07		
**p300/CBP 3000 ng/µl**	5.13	0.25	9	p=0.003
**p300/CBP 1000 ng/µl**	5.79	0.24	15.16	p<0.0000
**p300/CBP 250 ng/µl**	5.18	0.19	2.02	ns
**p300/CBP 50 ng/µl**	5.18	0.15	1.6	ns

Table S4. Statistical analysis of RNAi data for average number of offspring compared to the GFP control.

Treatment	Mean number	Standard error	Kruskal-Wallis test value	Significances
**Control (GFP)**	62.38	1.84		
**p300/CBP 3000 ng/µl**	11.11	0.81	317.24	p<0.000
**p300/CBP 1000 ng/µl**	11.84	1.17	306.36	p<0.000
**p300/CBP 250 ng/µl**	21.38	1.42	209.96	p<0.000
**p300/CBP 50 ng/µl**	23.02	1.31	184.17	p<0.000

Table S5. Reproductive parameters evaluated during the RNAi experiments including viviparous offspring determined by two-way ANOVA.

Treatment		Statistical parameters	Significances
**p300/CBP 3000 ng/µl**	Aphid line (control vs p300/CBP)	df=1 mean square=463.702 F=98.235	p<0.0000
	Age of reproduction	df=28 mean square=349.784 F=74.102	p<0.0000
	Aphid line x Age of reproduction	df=27 mean square=262.781 F=55.67	p<0.0000
**p300/CBP 1000 ng/µl**	Aphid line (control vs p300/CBP)	df=1 mean square=2462.32 F=596.994	p<0.0000
	Age of reproduction	df=30 mean square=204.834 F=49.662	p<0.0000
	Aphid line x Age of reproduction	df=16 mean square=203.719 F=49.392	p<0.0000
**p300/CBP 250 ng/µl**	Aphid line (control vs p300/CBP)	df=1 mean square=1829.640 F=373.094	p<0.0000
	Age of reproduction	df=30 mean square=289.921 F=59.120	p<0.0000
	Aphid line x Age of reproduction	df= 17mean square=167.724 F=34.202	p<0.0000
**p300/CBP 50 ng/µl**	Aphid line (control vs p300/CBP)	df=1 mean square=1173.761 F=242.996	p<0.0000
	Age of reproduction	df=30 mean square=297.476 F=61.584	p<0.0000
	Aphid line x Age of reproduction	df=20 mean square=132.424 F=27.415	p<0.0000

Table S6. Statistical analysis of RNAi data for average number of premature offspring compared to the GFP control.

Treatment	Mean number	Standard error	Kruskal-Wallis test value	Significances
**Control (GFP)**	0.43	0.05		
**p300/CBP 3000 ng/µl**	2.06	0.14	-185.09	p<0.000
**p300/CBP 1000 ng/µl**	2.98	0.17	-242.40	p<0.000
**p300/CBP 250 ng/µl**	1.89	0.24	-185.41	p<0.000
**p300/CBP 50 ng/µl**	1.57	0.24	-145.93	p<0.000

Table S7. Reproductive parameters evaluated during the RNAi experiments including premature offspring determined by two-way ANOVA.

Treatment		Statistical parameters	Significances
	Aphid line (control vs p300/CBP)	df=1 mean square=0.836 F=5.994	P=0.014
**p300/CBP 3000 ng/µl**	Age of reproduction	df=28 mean square=2.472 F=17.722	p<0.0000
	Aphid line x Age of reproduction	df=21 mean square=3.745 F=26.848	p<0.0000
	Aphid line (control vs p300/CBP)	df=1 mean square=8.954 F=41.950	p<0.0000
**p300/CBP 1000 ng/µl**	Age of reproduction	df=30 mean square=1.702 F=7.973	p<0.0000
	Aphid line x Age of reproduction	df=16 mean square=3.119F=14.614	p<0.0000
	Aphid line (control vs p300/CBP)	df=1 mean square=3.658 F=27.031	p<0.0000
**p300/CBP 250 ng/µl**	Age of reproduction	df=30 mean square=0.823 F=6.083	p<0.0000
	Aphid line x Age of reproduction	df=17 mean square=1.237 F=9.144	p<0.0000
	Aphid line (control vs p300/CBP)	df=1 mean square=0.842 F=6.774	p<0.009
**p300/CBP 50 ng/µl**	Age of reproduction	df=30 mean square=0.643 F=5.171	p<0.0000
	Aphid line x Age of reproduction	df=20 mean square=0.924 F=7.435	p<0.0000

Table S8. Statistical analysis of RNAi data for body weight [mg] compared to the GFP control (dsRNA concentration = 3000 ng/μl).

Time after injection [d]	Body weight p300/CBP [mg]		Body weight GFP control [mg]		Significances
	Mean	Standard error	Mean	Standard error	
**0**	1	0.02	0.92	0.02	ns
**3**	2.10	0.07	2.07	0.09	ns
**8**	2.86	0.09	2.89	0.07	ns

ns = not significant

Table S9. Statistical analysis of RNAi data for body size (length*width)[mm²] compared to the GFP control (dsRNA concentration = 3000 ng/μl).

Time after injection [d]	Body size p300/CBP [mm²]		Body size GFP control [mm²]		Significances
	mean	Std. error	mean	Std. error	
**3**	2.43	0.08	2.45	0.1	ns
**8**	3.07	0.07	3.07	0.06	ns

ns = not significant

Table S10. Statistical analysis of RNAi data for body color (grayscale) compared to the GFP control (dsRNA concentration = 3000 ng/μl).

Time after injection [d]	Body color p300/CBP - grayscale		Body color GFP control - grayscale		Significances
	Mean	Standard error	Mean	Standard error	
**3**	107.22	1.33	101.15	1.56	ns
**8**	75.59	2.4	95.41	0.95	p<0.01

ns = not significant

Fragment 1 of *A. pisum* p300/CBP mRNA, with primer positions underlined and indicated in bold (see Table S1).

CTGACCTTGGACCGTGAGTTCCCCGGACGCCGGTCACACTGGTGGCAATACGTCGATAAAGAACCAAAATTCTTGATAAAATTGTCAGCGAAAATTTTGTTTTTTATGCGTTTCGGTATAAATTTTTTACAAAAAATAGC
GTAAAACCATCCGTCGGACGAATTTCAGTAATTCCGGACGTTTGACGCGTTTGCCGGCCGTTTTCGCATAAACGGTGACAGCACAGCGCAACTATCGATAGTTGAGCGCTCCAAACAATCGTTGACCCGACTGCCGATG
GCTGCCCCCGTTCCCCGTATGTCTATCACGTCCCGTGAGTGCTTTATTTACATCTAGTTTTGTTGTCGAAAAATATATTTCCAGTGTCCGAATTCGCTGTTAATCCGTCGCGTTTACCGCATCGAACCGTCGCAGATCGTCG
GCGTACCGCGCAACGTGTCCAGGAACGCTTTCAGACGGTCCACCGAAGTGCCCAGGAACGGCCCTTATCAGCCGCAATCATCGGCTCGGATCACGTCAATTTCGGACACGGTCGGTTCTGCTTATGGCTGCCCATGTTA
CACACGAAACTCATCTTATGACTTGAATTCACCGCCCAGCTTCAAATATTAATTATTGTTGAACCATTTATCAGTCACAAAGAATTTTAACATGCAAGAATCGAGCTGAAAATAGAGACCTACAAATGAAATCTTCAATAA
TCAGCCTCCGACTTAGCATAACATGGCCCATATTACTGTATGGTGATATCACATACTGCTTAATTATGTGACTTAACTA**TGTTTAACTATTTTGTTTCTCAATTTG**

Fragment 2 of *A. pisum* p300/CBP mRNA, with primer positions underlined and indicated in bold (see Table S1).

AATCAGCCTCCGACTTAGCATAACATGGCCCATATTACTGTATAGTGATATCACATACTGCTTAATTATGTGACTTAACTATGTTTAACTATTTTGTTTCTCAATTTGAGTTTTTAAGAATTTGACATTGTAAAATAGGTTTA
GGTCATTGTGCAATTTTTAATATGTGATTATTGATACTAGTAACTTTTTACTAGGTCTTTTCTGTTTTTAAATTCTTAAACTAAATATGTCAGACCTATTGGTGGATAGCCCTCCGAACAAGAGGCCAAAGTTGGAAGATCC
ATTTCAAGGGTCTTCTGACTCATCTGGTGTCTATTCTAGCAATGAGATGTTTGATCTCGAGAATGATCTCCCTGATGAGCTTATTAGCACTACGTCTTGGAACCAGCAACCTGATATCAAGCCAAATCTTGGTCCTCAATTA
CCATTGCAAAATGGTATGATTGTTCAGCAAAATAATCCTCAACAACTTCAACAACCTAATATTCAACAACGATTAGTAGTCAGTCAACATCAGTTAGCTCACCATTTTATGGGTACTAAACATCTCGTGTCAGCGACTGGA
ATGTCTATGAATAAACCAATTAATCCAAACATGCCATCACCAAATGTTTTAGTTAGTAAAACTGGAGAACCGATGGTTGTAAATATGGGAAATGCAGGCATTAATTCCAATCAATTACAAAATAGTGTTAGTGGAATAAA
TGTAGCAAATGTAAATATTATGATGGGAAACAATTTGGTTAGTAATTCAATGCACAATCCTCATCATGGCATACCCCAGCAACAACAAAACGGACCTATGATGTTACGGAACATAGCCATGCATCATC**CACAACAAATTC
GCAACCAG**

Fragment 3 of *A. pisum* p300/CBP mRNA, with primer positions underlined and indicated in bold (see Table S1).

AGCAACAACAAAACGGACCTATGATGTTACGGAACATAGCCATGCATCATCCACAACAAATTCGCAACCAGACTCCTCATCAACTTCATAGTTTGCCAATTAATTCTCATAGGTTACAAGCTCCAATGGGTGCAATGAGT
CCAGTGAATAATTTCAATGCATACAACCAAAATAATCAACAAATTAATGTTGTCCAACAACAACCAATTGGTATTGTACGACAACCAGTACCGAGATTTAATGCCAACACTAATGGAATGTTAGTAGATACTCCGGTTCCT
CCTCAGTCTCAAGCAAATGTACGTCTTGGACCACAGCAAGTTAATATGGTCAATCAAAATGTAGTTGTACCACCTCAAGGAGTTCCCTCTGACCGTAGTCAAGCAAATCCAGAAAAACGAAAAATGATAACCCAACAGCT
TGTGCTATTATTACATGCACATCAATGTCAGAGACGAGATAATCAAGCTAATGGCGAAGTGAGACAATGTCGTTTGCAACATTGTAATACAATGAAAGGAGTCTTAGCTCATATGACCAATTGCTTAGCTGGTCGAAATT
GTAATGTAGCTCACTGTTCGTCATCTAGACAAATAATATCACATTGGAAACAATGTACTAGACCAGATTGTCCTATTTGCCAACCATTAAAAGCATCAAATAATCGCAATGCTTTAGCAAATAACACTACTCAAAATACTA
CTGGAATGAACGATATGAATTGTTATAGCACTGTAAATAATCAAGTGAACCAAGTACAGGGAGTCCCTAACAATGTTGGTATACGAACGGCTTTGCAATCTCAAATGCCACATCCTCCTAATAACAATGTGCTGACACCT
AATCAAAATACACGCGTATTAGTCC**CACATTCACAACCCCAGGTA**

Fragment 4 of *A. pisum* p300/CBP mRNA, with primer positions underlined and indicated in bold (see Table S1).

CTCAAATGCCACATCCTCCTAATAACAATGTGCTGACACCTAATCAAAATACACGCGTATTAGTCCCACATTCACAACCCCAGGTAACAACTACAATGAGTGCAGATCCCAGTACAAGTTCTGTAAATGAAATGCTGCAG
TCTGCTTCGAACACAACTGTTCCAGTTCCATCTAGCATTCAACAATCTGCGACAAATATTCCTAATTCTTTCATAATGACAACTAGTACTGATACAATGACTACTCAGATGAACAATCAAGCACAACCGAGTCAATCAATAC
CAGGAAATCACATTGAAAGTAAGGAATGGCATGAGTCTATTACACCTGAACTAAGAAATCATTTAGTGCATAAATTAGTTCAAGCAATTTTCCCAACATATGATCCTAAAGCAATGCTTGATAAACGTATGAATAATTTA
GTGGCTTATGCTAGAAAAGTAGAAGGTGATATGTACAATGTTGCGAACTCTAGGTCAGAATATTATCATAGGTTAGCCCAAACATTTTATAAAATACAAAAAGAGTTGGAAGAAAAAATGCAAAAGCGAAAAGAACAA
CAACAAATGCAACAGCAGCAATTGGGAACTCAGCAACCGACTAATAATATCAATGCACAATGCTTCCCAACTGCGTCTACACAAGGATTGCCTACTCAGCAGCAACCATCACAACCACAGCAGCAACAACCTTTACATCA
TACTTCAATTCAACCCAATCAGCAAAATTTGCCACAACAACAAGTAGTAACAACATCTGGTCTAATGAAAACACAAATG**TCAGACAGGGTACATTTTCCAA**

Fragment 5 of *A. pisum* p300/CBP mRNA, with primer positions underlined and indicated in bold (see Table S1).

TCAATTCAACCCAATCAGCAAAATTTGCCACAACAACAAGTAGTAACAACATCTGGTCTAATGAAAACACAAATGTCAGACAGGGTACATTTTCCAATCACTTCACCTGTACCTGGACCAAGTCCAAATCCAAATTTACCC
CCAACTACTTTTGGTTTGACCCAGCCACCTGTCCCAAGTCCAATCAACACTAGTCAGTTCCCATCTAACTCACCTATGATGTCAAACATATCTCAAGCTTCGTTCGCTCAACCTCAATTTCCAAATCAATCAATGAATCAAAT
GCCACAACAAATACATTTACAACAACAAGTTCAACAGCAACAACAAGTGCAACAGCAACAACAACAAGTACAACAGCAAGTTCAACAACAACAACACTTGAACAATCAAATGTTACAACCAAATAGACTTAATCAAATG
AATATGGAAAAAATGGTTAATGGACCAAGTTTGATAGCGAATAATACTGTGCCATCAACTACAAGTGTACCATCAATGATGCCCAATAGTATGGGTAAAGGCTATCCTAGATCAGAACCATCATCAATTAGTCATTCGAG
TCAGATGAATGCTATTGCTTCTGCTTTAGCACAAGATGACTCTCCCAAGGATGTTAAAGGGGAAGTAAAAGATGAGTATGATGATAGTAATGATGGAAAAGGTGCTTATGGAAAAGGTAAAATGAATTCTGAAACACC
GTCGGCATCTGTGAAATCTGAAATTAAACAAGAACGCGACGATAA**TGGTTCAAGTTCAAAGAAAATGG**

Fragment 6 of *A. pisum* p300/CBP mRNA, with primer positions underlined and indicated in bold (see Table S1).

AACACCGTCGGCATCTGTGAAATCTGAAATTAAACAAGAACGCGACGATAATGGTTCAAGTTCAAAGAAAATGGAAGTTAAAATTAAAGATGAACCTATGAGTCCTAAACCAAACATCACTACAGATATTAAACCATTT
GAGCCAACTACATCGGACAGTAAAACAGCCATAAAGAAATGTGTATTTAGGCCTGATGAACTTAGACAAGCCTTAATGCCTACTCTTGAAAAATTATACAAACAAGAACCTGAATCAGATCCTTTTAAACAGCCTGTTGA
TCCTCAAGCACTTAATATACCCGACTATTTCATAATCATAAAAAAGCCAATGGATTTATCAACAATTCGAGAAAAATTGAATACTGGTCAATTTTCTGACCCTTGGAGTTATGTTGATGATGTTTGGTTAATGTTTGAAAAT
GCTTGGCATTACAATAAGAAGTCTACAAAAGTATATAAATCTTGTACAAAACTTTCAGAAGTTTTTGAACAAGAAATTTATCCTGTTATGAGAAGTCTTGGTTATTGCTGTGGTCGTAAGTATACATTCAATCCGCAAGTA
CTTTGTTGTTTTGGAAAACAATTGTGTACTATTCCAAGGGATGCTAAATACTTCAGTTTTGAAAATAGATATATTTATTGTGTTAAATGCTTTAATGATATACCTGGTGATGCTGTAACATTGGGCGAAGACCCTACACAA
GCCCAACAAGTAATTAAAAAAGAACAATTTATGGAAATGAAAAATGATCATCTAGAATTGGAACCATTCATATTATGTACTCATTGTGGAAGAAAACTCCATCAAATATGTGTGCTGCACAACGAGAATATTAATCCACT
AGGATATGTTTGTGATAACTGTTTGAAGAAGCGCGGTGAAAAACGAAAAGATCATAAATTCAATGCTAAAAAATTGGCTATAACAAAATTAGGTACGTACATTGAAACAAGAGTGAACAATTTCCTTAAGAAAAAGAA
G**CAGGCGCTGGTGAAGTTGCT**

Fragment 7 of *A. pisum* p300/CBP mRNA, with primer positions underlined and indicated in bold (see Table S1).

ATTGGCTATAACAAAATTAGGTACGTACATTGAAACAAGAGTGAACAATTTCCTTAAGAAAAAAGAAGCAGGCGCTGGTGAAGTTGCTATACGTGTAGTGTCCAGTTCAGATAAAATAGTTGAAGTAAAACCTGGGAT
GCGTAGTCGCTTTGTTGATAATGGAGAGATGATAGGCGAATTTCCTTACAGGGCAAAAGCACTTTATGCATTTGAAGAAATTGATGGAACTGATGTTTGCTTCTTTGGTATGCATGTCCAAGAATATGGATCTGAAGCTC
CGTCACCCAATACAAGAAGAGTTTATATTGCATATTTAGATTCTGTCAACTTTTTCAGGCCCAAACAGTACAGAACGTATGTTTATCATGAAATATTATTGGGTTACCTTGATTATGTAAAACAATTAGGATATACTATGGC
TCACATTTGGGCATGCCCTCCATCTGAAGGTGACGATTACATATTTCATTGTCATCCACCTGAACAAAAAATACCAAAACCAAAACGATTACAAGAATGGTATAAGAAAATGCTAGATAAGGGTATAATTGAACGCATAA
TTCTGGACTATAAAGATATTTTAAAGCAAGCTATTGAAGATAAATTAAGTTCAGCAGCAGAATTACCTTACTTTGAAGGCGATTTTTGGCCAAATGCTTTAGAAGATAGCATTAAAGAACTTGACCAAGAAGAAGAACAG
AAGCGTAAACTAGCCGAGGCTGAAGAATCTGTTGAAATGTGTGTAAGTGATGAAGTTGAAATTGGCCCTGACGGAAAGAAAAAAGGTATGCAAAAGTCCAAAAAATCAAACAAATCTAA**GTCCAGCCAGAGAAAGA
GCA**

Fragment 8 of *A. pisum* p300/CBP mRNA, with primer positions underlined and indicated in bold (see Table S1).

GAAGTTGAAATTGGCCCTGACGGAAAGAAAAAAGGTATGCAAAAGTCCAAAAAATCAAACAAATCTAAGTCCAGCCAGAGAAAGAGCAGTTCAAAGAAAACTATGTTACCTCAAACTAGCAGTGATCTTTCTGCTAAA
ATTTTTGACAATATGGATAAACATAAAGAAGTGTTTTTTGTAATTAGATTGCATAGTGTACAGTCGGCTGCAGCGTTAGGAGCAATACAAGATCCAGATCCTTTAATCAGCTGTGATTTAATGGACGGTAGAGATGCATT
TTTGACCATGGCTCGAGAAAAACACTATGAATTTTCATCACTTAGGAGAGCAAAGTTTTCTTCAATGGCAATGTTGTATGAACTCCATAATCAAGGACAAGATAAATTTGTTTACACATGTAATAGTTGTAAATCACATGT
GGAAACCAGATACCACTGCACTGTTTGCGATGATTTTGATTTATGTATTACCTGTTACGATAAAGAAAAACATCCACATACCATGGAGAAATTGGGTCTTGATTTGGATGACGGATCATCTCCTGGTGACCAAAAACAAG
CAGATCCTCAAGAAGCTAGAAAATTATCTATCCGCCGGTGTATATTATCACTTGTACATGCTTGCCAATGTAGGGATGCAAATTGTCGCTTGGCAAGTTGTCAACGTATGAAAAGGGTGGTACAACATGTTAAACTATGT
AAAGTCAAATCTAATGGCAACTGTCCGATTTGTAAACAATATATTGCACTTTGCTTTCATCATGCTAAGTATTGTACTGAAGCTAAGTGTCCAGTGTTGTTCTGTCCAAATATGAAACACAAGATCAAACAGCAACAACTT
CAACA**AAGACTGCAACAAGCACAGC**

Fragment 9 of *A. pisum* p300/CBP mRNA, with primer positions underlined and indicated in bold (see Table S1).

TGTCCAGTGTTGTTCTGTCCAAATATGAAACACAAGATCAAACAGCAACAACTTCAACAAAGACTGCAACAAGCACAGCTATTAAGAAGACGTATGGCTATTATGAATACTGGTACTTTAGGGACGGAAAATACCACTC
CTGTTGTAGCAACACAACATATGTCTAATCAAACTAAAATGATACCTCATAAAGGGCCAGTTATGGTCCCAGGGAAACAAACACCGCCTGCCAATGTTTTACAAGTAGTTAAGCAAGTACAAGAAGAAGCGGCACGACA
ACATGATAACGTGGGATATGGAAAAGGTGGTCCTCAACAACAGATGCAACGACCACATATGCAGATCGCTCCCCCTGTTCCACAACAAGTCAATCAATGGGTTACTGAACCTAATTACAATCAAATGATGATGCAGCAG
CAACAACAGCAACAACAACAACAACAACAACAACAACAACAACAACAACAACAACAGCAGCAGCAACAGCAGCAGCAACAACAGCAGCAGCAACAACAAGTTCAACAGCAGCAGCAAAGACAAATGAACATTCAACC
TCAACAACAAATGATACTCAATAACCCACAAAATCCAATGCTTCAACAGCAACAAGTTATGACCTCCAGACCTCAAGGTTACCAACAAAACCCTAATCAACAAGTACCTAACCAATCTTTACAACAGTTACTAAATGCACT
GCGCACCAACACTGACAGTCCACCTGAACAGCAACAACGCCTAAGAGAGATTCTGAGGAGCAACCCACAGCTGTTGGCCCAATTCATTAGGAACCA**ACGACAAAGGCAAATGTTCC**

Assembled fragments of *A. pisum* p300/CBP mRNA. Accession number: MN734788

CTGACCTTGGACCGTGAGTTCCCCGGACGCCGGTCACACTGGTGGCAATACGTCGATAAAGAACCAAAATTCTTGATAAAATTGTCAGCGAAAATTTTGTTTTTTATGCGTTTCGGTATAAATTTTTTACAAAAAATAGCG
TAAAACCATCCGTCGGACGAATTTCAGTAATTCCGGACGTTTGACGCGTTTGCCGGCCGTTTTCGCATAAACGGTGACAGCACAGCGCAACTATCGATAGTTGAGCGCTCCAAACAATCGTTGACCCGACTGCCGATGG
CTGCCCCCGTTCCCCGTATGTCTATCACGTCCCGTGAGTGCTTTATTTACATCTAGTTTTGTTGTCGAAAAATATATTTCCAGTGTCCGAATTCGCTGTTAATCCGTCGCGTTTACCGCATCGAACCGTCGCAGATCGTCGG
CGTACCGCGCAACGTGTCCAGGAACGCTTTCAGACGGTCCACCGAAGTGCCCAGGAACGGCCCTTATCAGCCGCAATCATCGGCTCGGATCACGTCAATTTCGGACACGGTCGGTTCTGCTTATGGCTGCCCATGTTAC
ACACGAAACTCATCTTATGACTTGAATTCACCGCCCAGCTTCAAATATTAATTATTGTTGAACCATTTATCAGTCACAAAGAATTTTAACATGCAAGAATCGAGCTGAAAATAGAGACCTACAAATGAAATCTTCAATAAT
CAGCCTCCGACTTAGCATAACATGGCCCATATTACTGTATGGTGATATCACATACTGCTTAATTATGTGACTTAACTATGTTTAACTATTTTGTTTCTCAATTTGAGTTTTTAAGAATTTGACATTGTAAAATAGGTTTAGGT
CATTGTGCAATTTTTAATATGTGATTATTGATACTAGTAACTTTTTACTAGGTCTTTTCTGTTTTTAAATTCTTAAACTAAATATGTCAGACCTATTGGTGGATAGCCCTCCGAACAAGAGGCCAAAGTTGGAAGATCCATTT
CAAGGGTCTTCTGACTCATCTGGTGTCTATTCTAGCAATGAGATGTTTGATCTCGAGAATGATCTCCCTGATGAGCTTATTAGCACTACGTCTTGGAACCAGCAACCTGATATCAAGCCAAATCTTGGTCCTCAATTACCA
TTGCAAAATGGTATGATTGTTCAGCAAAATAATCCTCAACAACTTCAACAACCTAATATTCAACAACGATTAGTAGTCAGTCAACATCAGTTAGCTCACCATTTTATGGGTACTAAACATCTCGTGTCAGCGACTGGAATG
TCTATGAATAAACCAATTAATCCAAACATGCCATCACCAAATGTTTTAGTTAGTAAAACTGGAGAACCGATGGTTGTAAATATGGGAAATGCAGGCATTAATTCCAATCAATTACAAAATAGTGTTAGTGGAATAAATGT
AGCAAATGTAAATATTATGATGGGAAACAATTTGGTTAGTAATTCAATGCACAATCCTCATCATGGCATACCCCAGCAACAACAAAACGGACCTATGATGTTACGGAACATAGCCATGCATCATCCACAACAAATTCGCA
ACCAGACTCCTCATCAACTTCATAGTTTGCCAATTAATTCTCATAGGTTACAAGCTCCAATGGGTGCAATGAGTCCAGTGAATAATTTCAATGCATACAACCAAAATAATCAACAAATTAATGTTGTCCAACAACAACCAA
TTGGTATTGTACGACAACCAGTACCGAGATTTAATGCCAACACTAATGGAATGTTAGTAGATACTCCGGTTCCTCCTCAGTCTCAAGCAAATGTACGTCTTGGACCACAGCAAGTTAATATGGTCAATCAAAATGTAGTT
GTACCACCTCAAGGAGTTCCCTCTGACCGTAGTCAAGCAAATCCAGAAAAACGAAAAATGATAACCCAACAGCTTGTGCTATTATTACATGCACATCAATGTCAGAGACGAGATAATCAAGCTAATGGCGAAGTGAGAC
AATGTCGTTTGCAACATTGTAATACAATGAAAGGAGTCTTAGCTCATATGACCAATTGCTTAGCTGGTCGAAATTGTAATGTAGCTCACTGTTCGTCATCTAGACAAATAATATCACATTGGAAACAATGTACTAGACCAG
ATTGTCCTATTTGCCAACCATTAAAAGCATCAAATAATCGCAATGCTTTAGCAAATAACACTACTCAAAATACTACTGGAATGAACGATATGAATTGTTATAGCACTGTAAATAATCAAGTGAACCAAGTACAGGGAGTC
CCTAACAATGTTGGTATACGAACGGCTTTGCAATCTCAAATGCCACATCCTCCTAATAACAATGTGCTGACACCTAATCAAAATACACGCGTATTAGTCCCACATTCACAACCCCAGGTAACAACTACAATGAGTGCAGAT
CCCAGTACAAGTTCTGTAAATGAAATGCTGCAGTCTGCTTCGAACACAACTGTTCCAGTTCCATCTAGCATTCAACAATCTGCGACAAATATTCCTAATTCTTTCATAATGACAACTAGTACTGATACAATGACTACTCAGA
TGAACAATCAAGCACAACCGAGTCAATCAATACCAGGAAATCACATTGAAAGTAAGGAATGGCATGAGTCTATTACACCTGAACTAAGAAATCATTTAGTGCATAAATTAGTTCAAGCAATTTTCCCAACATATGATCCT
AAAGCAATGCTTGATAAACGTATGAATAATTTAGTGGCTTATGCTAGAAAAGTAGAAGGTGATATGTACAATGTTGCGAACTCTAGGTCAGAATATTATCATAGGTTAGCCCAAACATTTTATAAAATACAAAAAGAGTT
GGAAGAAAAAATGCAAAAGCGAAAAGAACAACAACAAATGCAACAGCAGCAATTGGGAACTCAGCAACCGACTAATAATATCAATGCACAATGCTTCCCAACTGCGTCTACACAAGGATTGCCTACTCAGCAGCAACC
ATCACAACCACAGCAGCAACAACCTTTACATCATACTTCAATTCAACCCAATCAGCAAAATTTGCCACAACAACAAGTAGTAACAACATCTGGTCTAATGAAAACACAAATGTCAGACAGGGTACATTTTCCAATCACTTC
ACCTGTACCTGGACCAAGTCCAAATCCAAATTTACCCCCAACTACTTTTGGTTTGACCCAGCCACCTGTCCCAAGTCCAATCAACACTAGTCAGTTCCCATCTAACTCACCTATGATGTCAAACATATCTCAAGCTTCGTTCG
CTCAACCTCAATTTCCAAATCAATCAATGAATCAAATGCCACAACAAATACATTTACAACAACAAGTTCAACAGCAACAACAAGTGCAACAGCAACAACAACAAGTACAACAGCAAGTTCAACAACAACAACACTTGAAC
AATCAAATGTTACAACCAAATAGACTTAATCAAATGAATATGGAAAAAATGGTTAATGGACCAAGTTTGATAGCGAATAATACTGTGCCATCAACTACAAGTGTACCATCAATGATGCCCAATAGTATGGGTAAAGGCT
ATCCTAGATCAGAACCATCATCAATTAGTCATTCGAGTCAGATGAATGCTATTGCTTCTGCTTTAGCACAAGATGACTCTCCCAAGGATGTTAAAGGGGAAGTAAAAGATGAGTATGATGATAGTAATGATGGAAAAGG
TGCTTATGGAAAAGGTAAAATGAATTCTGAAACACCGTCGGCATCTGTGAAATCTGAAATTAAACAAGAACGCGACGATAATGGTTCAAGTTCAAAGAAAATGGAAGTTAAAATTAAAGATGAACCTATGAGTCCTAA
ACCAAACATCACTACAGATATTAAACCATTTGAGCCAACTACATCGGACAGTAAAACAGCCATAAAGAAATGTGTATTTAGGCCTGATGAACTTAGACAAGCCTTAATGCCTACTCTTGAAAAATTATACAAACAAGAAC
CTGAATCAGATCCTTTTAAACAGCCTGTTGATCCTCAAGCACTTAATATACCCGACTATTTCATAATCATAAAAAAGCCAATGGATTTATCAACAATTCGAGAAAAATTGAATACTGGTCAATTTTCTGACCCTTGGAGTTA
TGTTGATGATGTTTGGTTAATGTTTGAAAATGCTTGGCATTACAATAAGAAGTCTACAAAAGTATATAAATCTTGTACAAAACTTTCAGAAGTTTTTGAACAAGAAATTTATCCTGTTATGAGAAGTCTTGGTTATTGCTG
TGGTCGTAAGTATACATTCAATCCGCAAGTACTTTGTTGTTTTGGAAAACAATTGTGTACTATTCCAAGGGATGCTAAATACTTCAGTTTTGAAAATAGATATATTTATTGTGTTAAATGCTTTAATGATATACCTGGTGAT
GCTGTAACATTGGGCGAAGACCCTACACAAGCCCAACAAGTAATTAAAAAAGAACAATTTATGGAAATGAAAAATGATCATCTAGAATTGGAACCATTCATATTATGTACTCATTGTGGAAGAAAACTCCATCAAATAT
GTGTGCTGCACAACGAGAATATTAATCCACTAGGATATGTTTGTGATAACTGTTTGAAGAAGCGCGGTGAAAAACGAAAAGATCATAAATTCAATGCTAAAAAATTGGCTATAACAAAATTAGGTACGTACATTGAAAC
AAGAGTGAACAATTTCCTTAAGAAAAAAGAAGCAGGCGCTGGTGAAGTTGCTATACGTGTAGTGTCCAGTTCAGATAAAATAGTTGAAGTAAAACCTGGGATGCGTAGTCGCTTTGTTGATAATGGAGAGATGATAGG
CGAATTTCCTTACAGGGCAAAAGCACTTTATGCATTTGAAGAAATTGATGGAACTGATGTTTGCTTCTTTGGTATGCATGTCCAAGAATATGGATCTGAAGCTCCGTCACCCAATACAAGAAGAGTTTATATTGCATATTT
AGATTCTGTCAACTTTTTCAGGCCCAAACAGTACAGAACGTATGTTTATCATGAAATATTATTGGGTTACCTTGATTATGTAAAACAATTAGGATATACTATGGCTCACATTTGGGCATGCCCTCCATCTGAAGGTGACGA
TTACATATTTCATTGTCATCCACCTGAACAAAAAATACCAAAACCAAAACGATTACAAGAATGGTATAAGAAAATGCTAGATAAGGGTATAATTGAACGCATAATTCTGGACTATAAAGATATTTTAAAGCAAGCTATTG
AAGATAAATTAAGTTCAGCAGCAGAATTACCTTACTTTGAAGGCGATTTTTGGCCAAATGCTTTAGAAGATAGCATTAAAGAACTTGACCAAGAAGAAGAACAGAAGCGTAAACTAGCCGAGGCTGAAGAATCTGTTG
AAATGTGTGTAAGTGATGAAGTTGAAATTGGCCCTGACGGAAAGAAAAAAGGTATGCAAAAGTCCAAAAAATCAAACAAATCTAAGTCCAGCCAGAGAAAGAGCAGTTCAAAGAAAACTATGTTACCTCAAACTAGCA
GTGATCTTTCTGCTAAAATTTTTGACAATATGGATAAACATAAAGAAGTGTTTTTTGTAATTAGATTGCATAGTGTACAGTCGGCTGCAGCGTTAGGAGCAATACAAGATCCAGATCCTTTAATCAGCTGTGATTTAATGG
ACGGTAGAGATGCATTTTTGACCATGGCTCGAGAAAAACACTATGAATTTTCATCACTTAGGAGAGCAAAGTTTTCTTCAATGGCAATGTTGTATGAACTCCATAATCAAGGACAAGATAAATTTGTTTACACATGTAAT
AGTTGTAAATCACATGTGGAAACCAGATACCACTGCACTGTTTGCGATGATTTTGATTTATGTATTACCTGTTACGATAAAGAAAAACATCCACATACCATGGAGAAATTGGGTCTTGATTTGGATGACGGATCATCTCCT
GGTGACCAAAAACAAGCAGATCCTCAAGAAGCTAGAAAATTATCTATCCGCCGGTGTATATTATCACTTGTACATGCTTGCCAATGTAGGGATGCAAATTGTCGCTTGGCAAGTTGTCAACGTATGAAAAGGGTGGTAC

AACATGTTAAACTATGTAAAGTCAAATCTAATGGCAACTGTCCGATTTGTAAACAATATATTGCACTTTGCTTTCATCATGCTAAGTATTGTACTGAAGCTAAGTGTCCAGTGTTGTTCTGTCCAAATATGAAACACAAGAT
CAAACAGCAACAACTTCAACAAAGACTGCAACAAGCACAGCTATTAAGAAGACGTATGGCTATTATGAATACTGGTACTTTAGGGACGGAAAATACCACTCCTGTTGTAGCAACACAACATATGTCTAATCAAACTAAA
ATGATACCTCATAAAGGGCCAGTTATGGTCCCAGGGAAACAAACACCGCCTGCCAATGTTTTACAAGTAGTTAAGCAAGTACAAGAAGAAGCGGCACGACAACATGATAACGTGGGATATGGAAAAGGTGGTCCTCAA
CAACAGATGCAACGACCACATATGCAGATCGCTCCCCCTGTTCCACAACAAGTCAATCAATGGGTTACTGAACCTAATTACAATCAAATGATGATGCAGCAGCAACAACAGCAACAACAACAACAACAACAACAACAAC
AACAACAACAACAACAACAGCAGCAGCAACAGCAGCAGCAACAACAGCAGCAGCAACAACAAGTTCAACAGCAGCAGCAAAGACAAATGAACATTCAACCTCAACAACAAATGATACTCAATAACCCACAAAATCCAA
TGCTTCAACAGCAACAAGTTATGACCTCCAGACCTCAAGGTTACCAACAAAACCCTAATCAACAAGTACCTAACCAATCTTTACAACAGTTACTAAATGCACTGCGCACCAACACTGACAGTCCACCTGAACAGCAACAA
CGCCTAAGAGAGATTCTGAGGAGCAACCCACAGCTGTTGGCCCAATTCATTAGGAACCAACGACAAAGGCAAATGTTCC

References

1. Sapountzis, P.; Duport, G.; Balmand, S.; Gaget, K.; Jaubert-Possamai, S.; Febvay, G.; Charles, H.; Rahbé, Y.; Colella, S.; Calevro, F. New insight into the RNA interference response against cathepsin-L gene in the pea aphid, Acyrthosiphon pisum: Molting or gut phenotypes specifically induced by injection or feeding treatments. *Insect Biochemistry and Molecular Biology* **2014**, *51*, 20–32.
